# Birth Experiences, Breastfeeding, and the Mother-Child Relationship: Evidence from a Large Sample of Mothers

**DOI:** 10.1177/08445621221089475

**Published:** 2022-04-07

**Authors:** Abi M. B. Davis, Valentina Sclafani

**Affiliations:** 1 School of Psychology, 4547 University of Lincoln, Lincoln, UK

**Keywords:** Breastfeeding, birth experiences, midwives, bonding, clinicians

## Abstract

**Background:**

It is a priority for public health professionals to improve global breastfeeding rates, which have remained low in Western countries for more than a decade. Few researchers have addressed how maternal perceptions of birth experiences affect infant feeding methods. Furthermore, mixed results have been shown in research regarding breastfeeding and mother-child bonding, and many studies are limited by small sample sizes, representing a need for further investigation.

**Purpose:**

We aimed to examine the relationship between subjective birth experiences and breastfeeding outcomes, and explored whether breastfeeding affected mother-infant bonding.

**Methods:**

3,080 mothers up to three years postpartum completed a cross – sectional survey.

**Results:**

Mothers who had more positive birth experiences were more likely to report breastfeeding their babies. Moreover, mothers who perceived their birth as more positive were more likely to breastfeed their child for a longer period (over 9 months) than those who had more negative experiences. In line with recent research, breastfeeding behaviours were not associated with reported mother-infant bonding.

**Conclusions:**

Mothers who reported better birth experiences were most likely to breastfeed, and breastfeed for longer. We find no evidence to suggest that feeding methods are associated with bonding outcomes.

## Introduction

Childbirth is a profound and life-changing event, and it is essential that mothers feel safe and supported throughout (World Health Organisation, 2018). Although all who bear children undergo the same basic biological events – a vaginal birth or a Caesarean section – its impacts on each mother are complex and unique (Beck & Watson, 2008). “Birth experiences” are characterised not only by medical procedures that may occur during the event, or a lack thereof, but also by less quantifiable feelings and circumstances, exclusive to a mother and her birth (Bell & Andersson, 2016). When a childbirth is perceived as negative, it can have short- and long-term psychological impacts, including postnatal depression and post-traumatic stress disorder (PTSD). Medical interventions and mode of delivery during childbirth have also been shown to affect breastfeeding initiation (Brown & Jordan, 2013; Saeed et al., 2011). Despite a focus on medical procedures across research, obstetric interventions are only one facet of childbirth. Several authors have argued that understanding a mother's *perceptions* of her experience is essential to interpret the outcomes of the event, and that these perceptions are not always based on medical complications or interventions (Beck & Watson, 2008; Muldoon et al., 2019). Indeed, Blüml and colleagues (2012) demonstrated that obstetric interventions are not universally regarded as negative by mothers, and studies have shown that perceptions of trauma surrounding childbirth can increase feelings of dissatisfaction, independent of emergency obstetric procedures (Fenaroli et al., 2019; Larsson et al., 2011). Further, a meta-analysis by Taheri et al. (2018) showed that positive labour experiences can *prevent* medical interventions, further suggesting a distinction between objective obstetric experiences and maternal perceptions of childbirth.

Although a relationship has been established between birth interventions and breastfeeding behaviours, the question of whether birth *perceptions* are associated with breastfeeding initiation and continuation remains under-researched. Moreover, most literature on the topic has focussed on ‘severe’ experiences and outcomes following birth, particularly post-traumatic stress disorder (e.g., Hairston et al., 2018; McDonald et al., 2011). Establishing whether negative birth experiences more broadly can affect infant feeding methods could be a key factor in improving breastfeeding rates. If they do, policymakers would be justified in extending their feeding support focus to all women who perceive their birth experience as negative, regardless of intervention status.

Additionally, it is important to determine any potential mother-child bonding consequences for women who choose not to, or are unable to, breastfeed. Better bonding as perceived by the mother, and secure infant attachment to the mother, are both associated with better cognitive, emotional, and behavioural outcomes for the child (De Cock, 2017; Richter, 2004). Systematic reviews have demonstrated a relationship between breastfeeding and secure infant attachment. However, the authors argue that evidence is mixed, and more studies with larger sample sizes are needed to draw conclusions about this relationship (Jansen et al., 2008; Linde et al., 2020). Recently, Hairston et al. (2019) found no relationship between feeding behaviours and bonding in Israeli mothers. The authors note that it would be beneficial to replicate this finding using other measures of bonding, and with the inclusion of more specific feeding groups, including a distinction between breast and combination feeding mothers. Further, significant cultural variability necessitates explorations across countries before claims of universality can be made (Keller, 2018). Given that the link between breastfeeding and bonding remains unclear, further explorations are warranted.

For practitioners to prioritise breastfeeding support in certain groups, with a view to improving global breastfeeding rates, we must first understand the experiences that affect a mother's likelihood of breastfeeding success. In the UK, approximately 81% of mothers in the UK initiate breastfeeding at birth, and by 5 weeks, only 55% continue to do so (Fox et al., 2015). This represents a significant discrepancy between planned or initiated feeding and later behaviours. Indeed, it may be that many more than 81% of mothers plan to feed their infants prior to birth (e.g., in 2003, Donath and Amir showed a 96.6% planned initiation rate in a cohort of 10,000 pregnant women in the UK). Thus, there is a clear need to establish predictors of breastfeeding cessation, to provide interventions designed to improve breastfeeding rates and outcomes.

Therefore, the first aim of this study was to investigate whether differences in birth experiences reported by mothers (comprising both perceptions of the birth and satisfaction with clinical care) were linked to different infant feeding methods. Further, we aimed to explore whether mothers in different feeding groups varied in their perceived relationships with their children. Our research questions were as follows: [RQ1] Do mothers’ feeding methods differ depending on their feelings about their birth experience? [RQ2] Is birth experience associated with the length of time an infant receives breast milk? [RQ3] Do mothers’ feelings about their relationship with their child differ depending on infant feeding method? [RQ4] Do mothers’ feelings about their relationship with their child differ depending on the length of time an infant received any breast milk?

## Methods

### Sample

The target population were mothers who had given birth in the last three years, with a view to later separating cohorts (those who gave birth under 12 months prior, and those who gave birth between 1–3 years prior). Following other researchers (e.g., Branjerdporn et al., 2019; Brown, 2014; Brown et al., 2014; DiTomasso et al., 2022), mothers were recruited through mother and baby forums online. As DiTomasso and colleagues note, as well as Wagg et al. (2019), thousands of mothers utilise online support groups for breastfeeding and parenting support, and these support groups can compensate for a lack of face-to-face or community services (Robinson et al., 2019). Over 60 parenting group administrators were contacted, and 37 of these groups advertised the survey.

Before data cleaning, 3,416 responses were collected. Some mothers indicated that their child was over 36 months old, and thus were excluded. Responses from duplicate IP addresses were also removed before data anonymisation. After data cleaning, 3,080 responses were retained. All participants were required to be mothers of children under 36 months of age, and no further exclusion criteria were applied. Maternal age ranged from 16–49 years, and the mean age of mothers was 28.17 years.

To assess infant feeding and the mother-child relationship, as well as infant feeding and birth experiences, an *a priori* G*Power analysis (Faul et al., 2009) for analyses of variance (ANOVA) was conducted. To achieve 95% power at an alpha of.05, and to detect a small effect (*f* = .10) with three feeding groups, a sample size of 1,548 (or 516 per feeding group) was required.

As we were interested in multiple categories within the population, we employed an oversampling strategy. This was required due to significant feeding method disproportions within the population, particularly for breastfeeding and long-term breastfeeding mothers (UNICEF, 2019).

### Measurements

#### Demographic information

Respondents reported maternal age, ethnicity, location, and the age category of their child (0–12 months or 12–36 months).

#### Perceptions of birth experiences

The Childbirth Perception Scale (CPS; Truijens et al., 2013) was used to measure participants’ birth experiences. This scale demonstrates good overall internal reliability (α = .82), and each subscale is adequately reliable (perception of delivery, α = .81; perception of the first postpartum week, α = .79). Participants respond using a 4-point Likert scale (fully agree to completely disagree). Scoring for the delivery subscale used in this manuscript ranges from 0–18. This scale was initially developed to assess experiences with vaginal births. However, Derya et al. (2019) have shown that it is reliable and valid when assessing childbirth experiences in samples of women who had both vaginal and caesarean births, and so the current study utilised this scale for all mothers.

#### Satisfaction with clinical care

Satisfaction with clinical care at the time of birth was measured using The Six Simple Questions (SSQ; Harvey et al., 2002). Reliability scores for this measure are high (α = .86), and the SSQ correlates with similar measures to an acceptable degree, *r* = .51 (Sawyer et al., 2013). The SSQ comprises six questions which are answered using a 7-point Likert scale, and scores range from 7–42. The questionnaire was adapted for the specific purpose of this study, and so the word “pregnancy” was replaced with “birth” where appropriate (e.g., “I would choose the same type of care for my next birth”). Authors have identified this method as acceptable when using the scale (Sawyer et al., 2013).

#### Maternal-infant relationship in the second and third postpartum year

To measure the quality of maternal-toddler relationships, mothers with children over one year of age completed the Child-Parent Relationship Scale (CPRS; Driscoll & Pianta, 1992), which has been validated as a measure for children in their second year and beyond (Simkiss et al., 2013). The CPRS is composed of 30 items measured using 5-point Likert scales, with options ranging from “definitely does not apply” to “definitely applies”. Scores are separated into three subscales: conflict (α = .83), closeness (α = .72), and dependence (α = .50).

#### Further information

Finally, participants indicated whether they fed their child with formula milk (FF), breast milk (BF), or a combination of the two (CF). If the child was not exclusively fed formula, mothers reported how long their child received any breast milk using predetermined categories (see Table S1 for categorisations and incidence).

To measure attachment in the first postpartum year, mothers completed the Maternal Postnatal Attachment Scale (MPAS; Condon & Corkindale, 1998). However, we did not include mothers in their first postpartum year in our analyses of breastfeeding behaviours, due to the potential for incorrect categorisations (e.g., a mother with a three-week-old infant could report “exclusively breastfed for under four weeks” and continue breastfeeding for two years).

Responses were also collected for the Hospital Anxiety and Depression Scale (HADS) (Zigmond & Snaith, 1983). However, this scale was not directly relevant to our research focus on this instance, and was therefore only used as a control variable. The HADS has been used extensively in research, and it has been shown to be reliable and valid across multiple populations (α = .89, *r* = .83–.86; Boxley et al., 2016).

### Data collection

Data were collected over 6 consecutive weeks in April 2020. The questionnaire was hosted through Qualtrics (www.qualtrics.com). After giving consent, respondents completed the questionnaire.

All participation was voluntary and not incentivised, and participants were free to modify their answers or exit the survey at any time. The survey took approximately ten minutes to complete. All data were kept in a secure, password-protected university OneDrive account to ensure confidentiality.

This study was granted ethical approval by the university's ethics committee (Approval number: 1920314). All ethical requirements for work with human participants were followed. Participants were introduced to the study prior to participation, and were debriefed upon completion.

### Data analysis

Data were analysed using SPSS version 24.0 (SPSS Inc., Chicago, IL, USA), R (R Foundation for Statistical Computing), and JASP (Love et al., 2019). One-way, between-subjects ANOVAs were conducted to determine whether childbirth perceptions as measured by the CPS and the SSQ differed across infant feeding method groups (exclusive breastfeeding, combination, or exclusive formula).

To assess feeding group differences for mother-child bonding as measured by the CPRS, one-way, between-subjects ANOVAs were performed for each of the CPRS subscales (“conflict”, “closeness”, and “dependence”).

A Bayesian ANOVA was conducted as a confirmatory test for findings related to breastfeeding and bonding outcomes, and a linear regression was conducted to determine the relationship between SSQ and CPS scores.

Seventeen mothers reported that they exclusively breastfed their child, without formula inclusion, for 1–9 months. As guidance states that mothers should feed their infants either breast, formula milk, or a combination of both, for at least 12 months, these mothers may have mis-categorised themselves or changed feeding methods. To avoid the inclusion of miscategorised data, we excluded these responses from our analysis.

All items from the CPS were reverse coded during analysis for clarity, so that higher scores indicated better experiences.

## Results

Participants were all female as assigned at birth. Descriptive statistics for participants are presented in Table 1.

**Table 1.** Demographic Characteristics of the Sample.

Measure	Descriptive	*N*	%	*M*	*SD*
Age		3,041		28.16	5.63
Ethnicity	White	2,862	94.8%		
	Mixed or multiple groups	60	2.0%		
	Asian	46	1.5%		
	Other	27	1.2%		
	Black or African	13	0.4%		
Child age	<12 months	1,178	39.0%		
	12 months-3 years	1,842	61%		
Feeding method	Exclusive formula	868	37.0%		
	Exclusive breastmilk	819	34.9%		
	Combination feeding	660	28.1%		

Most mothers fed their children formula milk or breastfed their children for over 12 months (representing 66.9% of the total sample). Feeding categories and reported incidence are illustrated in Table S1 in the Supporting Information file.

### Preliminary/exploratory analyses

#### Breastfeeding and age differences

Analyses exploring age differences across feeding groups revealed a significant main effect, *F*(2, 2,334) = 102.08, *p* < .001, Cohen's *f* *=* .69. Those who exclusively breastfed their infants were the oldest group in the cohort (*M* = 30.22 years, *SD* = 5.43). The CF group younger than BF (*M* = 28.50 years, *SD* = 5.65, *f* = .36), and younger still were the FF group (*M* = 26.45 years, *SD* = 5.21, Cohen's *f* vs. BF = .69; *f* vs. CF = .29). All *p*s < .001.

#### Satisfaction with care and birth experiences

When controlling for age, depression, and anxiety, and CPRS subscales, a multiple regression showed that satisfaction with care (SSQ) significantly predicted better birth experiences (CPS), *F*(6, 1,281) = 63.00, *p* *<* *.*001), with a large effect (Cohen's *d* = 1.00).

### Results for Rq1–4

#### Infant feeding behaviours and birth experiences [RQ1]

**Figure 1.**
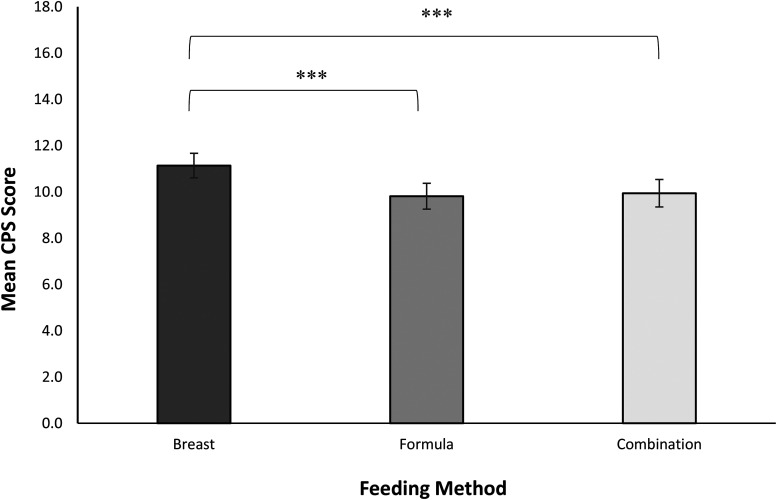
Responses for the CPS by feeding method. Responses are expressed in terms of mean ratings on the 0–4-point Likert scale. Error bars represent 95% confidence intervals. *** *p* < .001.

The data for this analysis are summarised in Figure 1.

First, we compared feeding groups and CPS birth experiences. Results showed a significant main effect of feeding method*, F*(2, 2,235) = 26.94, *p* < .001, Cohen's *f* = .30. Post-hoc Tukey HSD comparisons revealed that the ‘BF’ group reported significantly higher scores on the CPS, representing a better birth experience (*M* = 11.13, *SD* = 3.94), in comparison with both the ‘CF’ group (*M* = 9.94, *SD* = 3.82, *p* < .001, *f* = .23) and the ‘FF’ group (*M* = 9.81, *SD* = 3.89, *p* < .001, *f* = .28). There were no significant differences in CPS scores between the formula and combination feeding groups (*p* = .802).

For satisfaction with clinical care, a main effect of feeding type was found, *F*(2, 2,297) = 13.42, *p* < .001, Cohen's *f* = .31. Those who exclusively breastfed reported higher levels of satisfaction with clinical care (*M* = 32.78, *SD* *=* 8.22) in comparison with both those who combination fed (*M* = 30.59, *SD* *=* 8.51, *f* = .08) and those who formula fed their children (*M* = 31.21, *SD* = 8.62, *f* = .29), both *p*s < .001. In this case, only the ‘breast versus formula’ comparison yielded a practically significant effect size. There was no significant difference in satisfaction with care between the ‘combination’ and ‘formula’ feeding groups (*p* = .337).

Given that birth experiences precede infant feeding, we conducted a multinomial logistic regression to establish the predictive and clinically relevant nature of birth experiences. Results are outlined in Table 2. First, we compared the intercept only model with a model in which the CPS was a predictor variable, and confirmed that the CPS model was significantly better, *χ^2^*(2) = 53.29, *p* < .001.

**Table 2.** Multinomial Logistic Regression Results.

Feeding method	Coefficients		Odds ratios		*p*
-	Intercept	CPS (delivery)	Intercept	CPS (delivery)	
Combination	.64 (0.16)	−.08(.01)	1.90	.92	<.0001
Formula	.99 (0.15)	−.09(.01)	2.69	.92	<.0001

Reference category: breast; values in brackets represent the standard error; p values for Wald's z-test.

The odds ratios outlined in Table 2 illustrate that a decrease in one unit (or point) on the CPS scale was associated with an 8% increase in the likelihood of formula or combination feeding (respectively) in comparison with breastfeeding. When comparing mean scores from each feeding group, the data represent a 9.5% actual increase in the odds of combination feeding, and a 10.5% decrease in the likelihood of formula feeding.

#### Birth experiences and infant feeding durations [RQ2]

**Figure 2.**
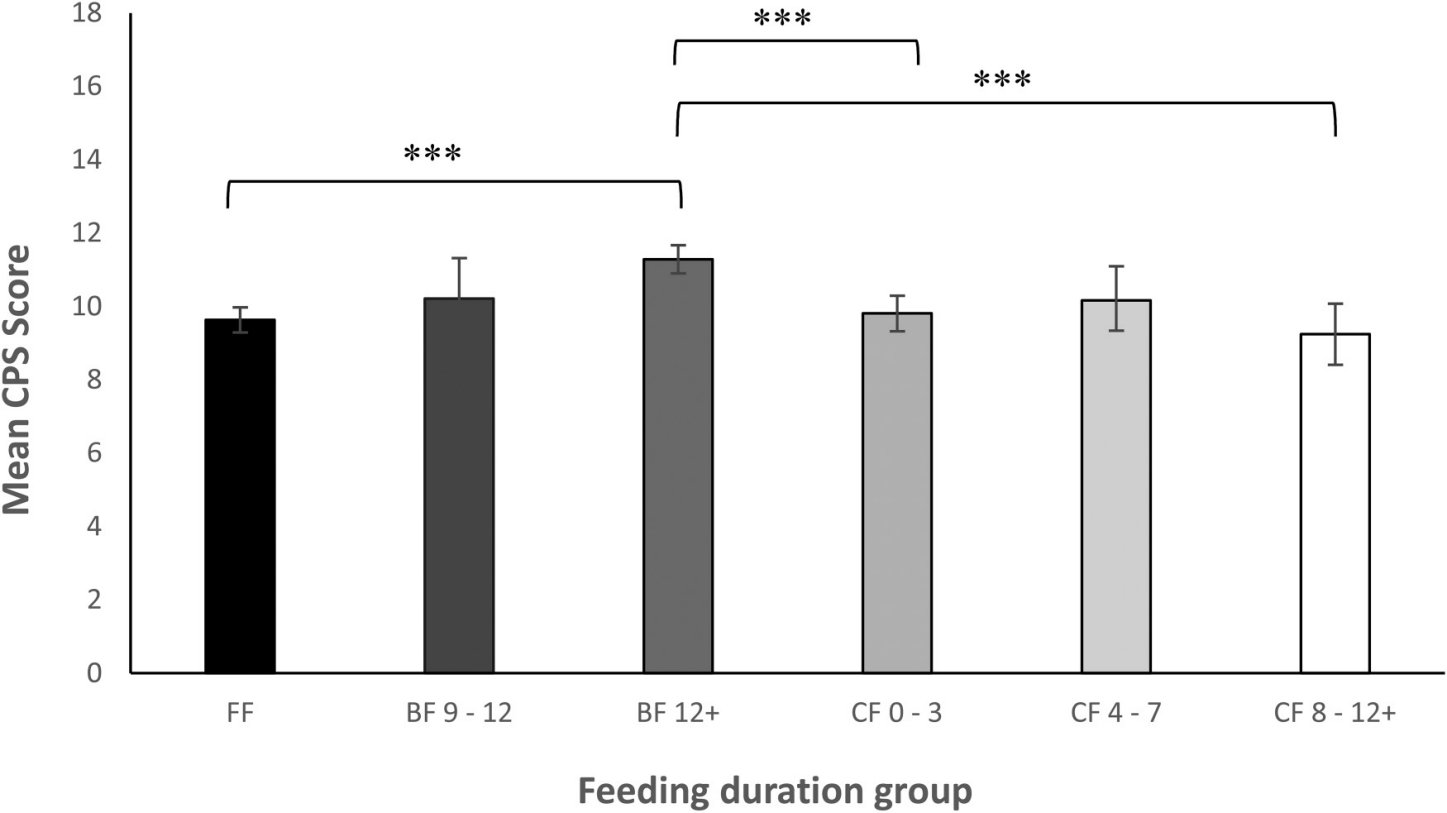
Responses for the CPS by feeding duration. FF = formula fed; CF = combination fed (‘mo’ represents number of months the child received any breast milk); BF = breastfed. Responses are expressed in terms of mean ratings on the 0–4-point Likert scale. Error bars represent 95% confidence intervals. *** *p* < .001.

The data for this analysis are summarised in Figure 2.

For birth experience (CPS) scores and ‘feeding duration’ groups, a significant main effect was found, *F*(5, 1,352) = 9.93, *p* < .001, Cohen's *f* = .38. Post-hoc tests showed that the only ‘durations’ group which differed significantly from others for CPS scores was the ‘exclusive breastfeeding->12 months’ group, which differed from the formula group (*p* < .001), the ‘CF- 1–3 months’ (*p* < .001) and ‘CF- 8–12+ months’ (*p* = < .001) groups. Cohen's *f* effect sizes and significance varied for comparisons between this group and other feeding duration groups. (See Table S2 for between-group comparisons and more information.)

Next, we compared infant feeding duration groups’ satisfaction with clinical care as measured by the SSQ. A significant main effect was found, *F*(5, 1,377) = 5.05, *p* < .001, Cohen's *f* = .40. Again, the only group which significantly differed from others was the ‘breastfeeding- >12 months’ group (*M* = 32.63) Mothers in this category differed from the ‘formula’ group (*M* = 30.64, *p* = .011, *f* = .28) and the ‘combination- 1–3 months’ group. (*M* = 28.83, *p* < .001, *f* = .36). Table S3 shows detailed between-group comparisons and effect sizes.

We next sought to identify how age might influence the relationship between breastfeeding and birth experiences. As we found an association between the three feeding methods and age, the assumption of independence between independent variables for ANCOVA analysis was violated (for a discussion, see Owen & Froman, 1998). Therefore, we could not include age as a covariate in an ANCOVA model. Instead, we conducted linear regressions to establish whether age predicted CPS birth experience scores for each individual feeding group, respectively. No significant associations were found in these individual models, or a model which included all three groups (all *p*s > .05), suggesting that age did not influence this relationship.

#### Breastfeeding and the child-parent relationship in mothers with infants 1–3 years old [RQ3]

First, we treated ‘feeding type’ as a trichotomous variable, comparing formula, breast, and combination feeding groups. Responses on the CPRS did not significantly differ based on feeding type for either the ‘conflict’, *F*(2, 1,398) = 1.94, *p* = .144, Cohen's *f* = .02, or ‘closeness’ subscales, *F*(2, 1,379) = 2.26, *p* = .105, Cohen's *f* = .02. A statistically significant main effect of feeding type for the dependence subscale was found, *F*(2, 1,394) = 3.54 *p* = .029, with breastfeeding mothers scoring higher (*M* = 3.15, *SD* = .60) than mothers who fed their infants formula milk (*M* = 3.05, *SD* = .62, *p* = .022). However, the effect size was negligible (Cohen's *f* = .06). The combination and formula feeding groups did not significantly differ (*p* = .565), nor did the breast and combination groups (*p* = .219).

#### Breastfeeding duration and mother-child relationship outcomes [RQ4]

Next, we explored whether mothers reported differences in their perceived relationship with their child based on the amount of time their infant received breast milk. Analyses showed no main effect for the CPRS ‘conflict’, *F*(7, 1,390) = .99, *p* = .418 or CPRS ‘dependence’ subscales, *F*(5, 1,388) = 1.64, *p* = .145. A significant main effect was found for the ‘closeness’ subscale, *F*(5, 1,371) = 2.57, *p* = .025, showing that the ‘combination feeding- received breast milk for 0–3 months’ group received higher closeness scores. However, post-hoc Tukey tests did not show any statistically significant group differences. Further, effect sizes for between-group comparisons were statistically negligible, with Cohen's *f* effect sizes ranging from .00–.04. No significant between-group differences were found when assessing differences in our three ‘combined’ feeding groups (as outlined under the ‘Data analysis’ subheading) for scores on the CPRS subscales (‘closeness’, *p* = .365; ‘conflict’, *p* = 409; ‘dependence’, *p* = .507).

### Bayesian confirmation

To test the likelihood of not rejecting the null hypothesis given the data, a Bayes Factor (BF) ANOVA was conducted, with breastfeeding groups as fixed factors, and the CPRS subscales as dependent variables. This analysis supported our previous ANOVA results, showing that the likelihood of the null hypothesis given the data for the ‘dependence’ subscale was 3.78 times greater than for the alternative hypothesis. For the ‘conflict’ subscale, the likelihood of the null hypothesis was 17.94 times greater, and for ‘closeness’, 13.04 times greater. The former represents *moderate evidence* for the null hypothesis, and the latter two, *strong evidence* (Lee & Wagenmakers, 2014).

## Discussion

Researchers have shown that birth interventions may prevent breastfeeding initiation (Brown & Jordan, 2013; Saeed et al., 2011). However, literature which investigates a mother's *perceptions* of her birth experience and subsequent breastfeeding behaviours remains scant. We sought to address this gap in the literature by surveying a large sample of mothers, focussing on their subjective childbirth experiences. Moreover, we investigated whether mother-infant bonding was associated with different infant feeding methods.

Findings showed that mothers who were more satisfied with clinical care at the time of birth, and who perceived their birth experiences as more positive, were more likely to report breastfeeding their children. Better satisfaction and more positive perceptions were also associated with breastfeeding past the child's first birthday. Consistent with Hairston et al.’s (2019) findings, which showed that feeding type was not associated with bonding, we also found no relationships between infant feeding method and mother-child relationship outcomes.

### Birth experiences and breastfeeding

Our data suggest that a positive birth experience is associated with the care received by healthcare staff, therefore highlighting the importance of good clinical care for maternal birth satisfaction. This is in line with previous investigations (e.g., Blüml et al., 2012). Our findings are also in line with Goyal et al. (2014), who demonstrated that better hospital care can facilitate breastfeeding initiation. Given this relationship between clinical care and overall perceptions of birth experiences when exploring subjective experiences of childbirth, researchers should be cautious in utilising assessment tools which focus solely or primarily on obstetric interventions or surgical procedures.

Our analyses showed that mothers who had better birth experiences were more likely to breastfeed their children, and that those who breastfed their children for over 12 months had better birth experiences than formula feeding mothers and some combination feeding mothers. Odds ratios revealed a 9.5% increase in the likelihood of combination feeding for our cohort, and a 10.5% increase in the likelihood of formula feeding, based on lower average birth experience scores when compared with mothers who exclusively breastfed. Results from this study suggest that childbirth experiences may be an area of interest for predicting breastfeeding initiation. As suggested by Beck and Watson (2008), negative birth experiences can impede breastfeeding initiation and continuation. In the short-term, perceived birth trauma, obstetric interventions, and physical pain can prevent initiation, leading women to introduce formula milk. In the longer term, negative birth experiences can increase the risk of flashbacks, PTSD, low postpartum mood, and detachment from the child. Klein and colleagues (2014) also discussed how negative birth experiences can contribute to feelings of inadequacy in mothers to care for and breastfeed their child, further increasing the likelihood of formula introduction.

As well as emotional consequences, the introduction of formula has various associated health risks. Breastfeeding decreases the risk of several illnesses for both mother and child, including maternal breast and ovarian cancers, and child allergies, asthma, and gastric issues (for an overview, see Davis et al., 2021; Dieterich et al., 2013). A recent meta – analysis conducted by Su et al. (2021) demonstrated a 23% reduction in the likelihood of childhood leukaemia when an infant was breastfed or occasionally breastfed versus being formula fed, and a 23% decreased risk between the longest and shortest breastfeeding durations. It is clear based on evidence that there is a significant need to improve breastfeeding rates in the United Kingdom. By understanding which factors influence feeding behaviours, interventions can be applied with a view to facilitating breastfeeding for mothers who wish to do so.

As well as the impact of a negative birth on breastfeeding directly, several factors might contribute to both birth experiences and longer-term breastfeeding, including socioeconomic status (SES) and access to resources. Mothers who have fewer economic barriers are more likely to have successful breastfeeding journeys than those with lower socioeconomic backgrounds (Gebrekidan et al., 2020; Oakley et al., 2013). We did not include SES as a variable in the current study, and therefore we cannot make any assumptions about whether mothers’ SES had an influence on their birth experiences, or their decision to breastfeed. Future studies should control for this variable and explore whether higher SES and access to resource can affect mothers’ birth experience and breastfeeding behaviours.

Maternal age may also contribute to both better birth experiences and longer-term breastfeeding (Oakley et al., 2013). However, our findings suggest that, independent of their age, longer-term breastfeeding mothers perceived their birth experiences as more positive. Interestingly, although no age effect was found, longer-term breastfeeding mothers were the oldest group in the cohort, on average four years older than formula feeding mothers. As shown by Smith and colleagues (2012), younger mothers can have more negative perceptions of breastfeeding and suffer more social stigma than older mothers (Smith et al., 2012). Although beyond the specific scope of our study, our findings highlight the importance of future breastfeeding research and support for younger mothers, who should be considered an at-risk group for early breastfeeding cessation.

Although we found that breastfeeding mothers and those who combination fed for 4–7 months did not differ in their birth experiences, this finding may be an issue of statistical power, rather than a true lack of differences (i.e., the relatively small sample of *n* = 70 was sufficient to detect medium, but not small, differences between groups). Further research is necessary to determine any differences in experiences between longer-term breastfeeding and combination feeding mothers. More generally, very few explorations have assessed the behaviours of mothers who continue to offer breast milk alongside formula milk for longer periods, representing a clear need for further inquiry.

Formula feeding mothers and those who combination fed their child for 0−3 months reported experiencing lower satisfaction with care at the time of birth than breastfeeding mothers. This finding suggests that it is critical for care providers to ensure support throughout the birth experience to facilitate breastfeeding. A wealth of qualitative research has shown that care from midwives can be crucial in the formative stages of breastfeeding initiation (see Battersby, 2014, for an overview), and midwives in Great Britain are experiencing staffing issues, heavy workloads, and a lack of time to focus on person – centred care (Edwards et al., 2018; Royal College of Midwives, 2018).

Our results suggest that facilitating good birth experiences and prioritising adequate patient support should be deemed vital for healthcare staff, for the wellbeing of mothers (Leeds & Hargreaves, 2008), and for the potential benefits it may offer for breastfeeding initiation and continuation.

### Breastfeeding and mother-child bonding

Next, we explored whether feeding methods were associated with the mother-child relationship. In line with Hairston et al. (2019), we found no bonding differences between mothers who used different feeding methods. Further, the length of time an infant received breast milk was not associated with any differences in bonding.

Studies regarding the mother-child relationship and feeding methods have yielded mixed results and are often complicated by social and scientific assumptions about breastfeeding and mother-infant bonding. A body of scientific and lay literature on the topic is predicated on the notion that breastfeeding facilitates bonding, and these assumptions have informed public health policies globally (e.g., WHO, 2013, National Health Service, 2020). Despite this, only limited and often underpowered studies have shown such an association (Jansen et al., 2008; Linde et al., 2020). Our study aligns with the few well-powered empirical studies which do not show this association (following Hairston et al., 2019, and Else-Quest et al., 2003). We therefore extended previous findings to include mothers of 1–3-year-old infants, showing a consistent null effect of feeding type on bonding outcomes across the first three years of an infant's life.

This finding supports early understandings of attachment and bonding. In his seminal study, Harlow & Harlow (1962) showed that in rhesus macaques, infant monkeys who had warm, comforting terry cloth mothers fared better behaviourally than those who had wire mothers, even when the monkeys consumed the same amount of milk. Harlow concluded that normal development can only occur when comfort is available to an animal, and this was true regardless of the availability of milk. Harlow's studies were the basis for Bowlby's widely accepted ethological theory of attachment, which relies on the idea that that attachment is not inherently based on physiological feeding needs, but on responsive caregiving (see Bretherton, 1992 for a discussion). Our finding that breastfeeding was not associated with bonding is consistent with these theoretical arguments, suggesting that bonding is not necessarily a product of feeding method.

It is important to note that there is a distinct lack of literature which explores the relationship between perceived bonding and objective measures of attachment. Therefore, although we show no relationship between breastfeeding behaviours and *perceived* bonding, it remains important to continue work exploring breastfeeding and attachment security (Linde et al., 2020). Nevertheless, our findings add to a growing body of literature which suggests that whilst breastfeeding remains important for global health, it may not be essential for mother-child bonding.

### Limitations

This research was exploratory, and many covariates were not accounted for. These included parity status, infant gender differences, maternal socio-economic status, and indeed, obstetric interventions. All these factors have been shown to influence birth experiences or breastfeeding (Brown & Jordan, 2013; Goyal et al., 2017; Oakley et al., 2013; Rijnders et al., 2008). Further studies are needed to determine the primary reasons for the relationship between childbirth perceptions and breastfeeding outcomes. Here, we argue that perceptions of a birth experience may not be due to obstetric interventions alone. To confirm this, an exhaustive study which encompasses all aspects of a birth experience is necessary.

The retrospective nature of this study may be a limitation. However, a longitudinal study conducted by Takehara and colleagues (2014) showed that mothers remember their birth experiences (both subjective perceptions and objective circumstances) clearly and accurately across at least a five-year period, likely due to the significance of the event. Mothers who participated in the current study were a maximum of three years postpartum, thus falling within Takehara's scope for accurate recollection.

Most mothers in the sample self-reported as white (94.8%). Caution should be applied when generalising these findings, though we provide support for Hairston et al. (2019)'s data showing a lack of association between birth experiences and bonding in Israeli mothers. It is critical that the experiences of mothers of colour are investigated, given the unique challenges that they can face during childbirth, including an increased risk of death (Knight et al., 2019; Lister et al., 2019). Future research should consider the experiences of underrepresented groups as a matter of priority.

Finally, mothers were recruited in April 2020, during the COVID-19 pandemic. Although our primary analyses did not include mothers who gave birth in the preceding 12-month period, some analyses (e.g., the effects of satisfaction with care on birth perceptions) included mothers who may have given birth at the start of the pandemic. Although it is beyond the scope of this particular project to account for the effects of the pandemic on birth experiences, this remains an important area for exploration.

### Implications for practice and future research

The results from this study support research which shows that staff can influence birth experiences in several ways. Individualised care and continuity of care can be important for a mother's perception of her experience (Dahlberg & Aune, 2013), and perceptions of choice in a care context can promote better subjective experiences (Cook & Loomis, 2012; Jelly et al., 2020). It is important for future researchers to explore the complex interplay between subjective perceptions of birth, feelings and perceptions around clinical care at the time of birth, and objective obstetric experiences. This in turn will help obstetricians to better understand the factors which improve childbirth perceptions, regardless of obstetric intervention status, and to ultimately empower women throughout childbirth.

## Conclusions

Global breastfeeding rates in 2020 represent a significant public health issue. As well as the risks associated with breastfeeding cessation, mothers who wish to breastfeed but do not can be vulnerable to feelings of guilt related to their experiences (Ayton et al., 2019). By identifying women who perceive their childbirth as a negative event, practitioners can provide early support, and thus alleviate, potential barriers to breastfeeding. Nevertheless, if a mother chooses not to, or cannot, breastfeed, we did not find evidence to suggest that their relationship with their child will be affected.

## Supplemental Material

sj-docx-1-cjn-10.1177_08445621221089475 - Supplemental material for Birth Experiences, Breastfeeding, and the Mother-Child Relationship: Evidence from a Large Sample of Mothers Click here for additional data file. Supplemental material, sj-docx-1-cjn-10.1177_08445621221089475 for Birth Experiences, Breastfeeding, and the Mother-Child Relationship: Evidence from a Large Sample of Mothers by Abi M. B. Davis and Valentina Sclafani in Canadian Journal of Nursing Research
