# Supplementary material for: Birth Experiences, Breastfeeding, and the Mother-Child Relationship: Evidence from a Large Sample of Mothers
Source: Can J Nurs Res. 2022 Apr 7;54(4):518–29. doi: 10.1177/08445621221089475 (PMC9597165; doi:10.1177/08445621221089475)
Supplement: sj-docx-1-cjn-10.1177_08445621221089475 - Supplemental material for Birth Experiences, Breastfeeding, and the Mother-Child Relationship: Evidence from a Large Sample of Mothers [file sj-docx-1-cjn-10.1177_08445621221089475.docx]

**Supporting information**

For ‘feeding duration’ groups, we conducted a power analysis to determine the sample size needed to detect a medium effect (*f* = 0.25). We chose a medium effect due to the potential for very few mothers to be categorised into certain groups, based on breastfeeding statistics provided by the United Nations International Children’s Emergency Fund (UNICEF, 2019). Due to these small groups, a very large overall sample would be needed to identify these mothers, and a larger sample still to detect a small effect. To achieve 95% power at an alpha of 0.5, a minimum of 33 participants per group was required. Table S1 illustrates group incidence for each ‘feeding duration’ category.

**Table S1. Descriptive statistics for feeding duration groups**

| **‘Feeding duration’ group** | ***N*** | **%** |
| --- | --- | --- |
| Formula (FF) | 501 | 35.5% |
| CF- Breast 0-3 mo | 268 | 19% |
| CF- Breast 4-7 mo | 72 | 5.1% |
| CF- Breast 8-12+ mo | 80 | 5.7% |
| BF- stopped 9- 12 mo | 48 | 3.4% |
| BF- stopped >12 mo | 443 | 31.4% |
|  | Total = 1,412 | Total = 100% |

**Table S2. Between-group comparisons and effect sizes for feeding method and CPS scores**

| **Feeding type** |  | **Mean Difference** | **Std. Error** | **Sig.** | **95% CI** | **Cohen’s *f*** |
| --- | --- | --- | --- | --- | --- | --- |
| FF (1) | 2 | -.525 | .610 | .955 | [-2.27, 1.22] | 0.05 |
|  | 3 | -1.652 | .261 | **< .001** | [-2.40, -0.91] | **0.34** |
|  | 4 | -.175 | .300 | .992 | [-1.03, 0.68] | 0.03 |
|  | 5 | -.584 | .500 | .852 | [-2.01, 0.84] | 0.06 |
|  | 6 | .390 | .475 | .964 | [-0.97, 1.74] | 0.04 |
| BF 9-12(2) | 3 | -1.127 | .614 | .443 | [-2.88, 0.63] | 0.10 |
|  | 4 | .350 | .631 | .994 | [-1.45, 2.15] | 0.02 |
|  | 5 | -.059 | .748 | .100 | [-2.19, 2.08] | 0.04 |
|  | 6 | .915 | .731 | .85 | [-1.17, 3.00] | 0.07 |
| BF 12+(3) | 4 | 1.477 | .308 | **< .001** | [0.60, 2.36] | **0.25** |
|  | 5 | 1.038 | .505 | .281 | [-0.37, 2.51] | 0.11 |
|  | 6 | 2.042 | .480 | **< .001** | [0.67, 3.41] | **0.23** |
| CF 0-3(4) | 5 | -.409 | .527 | .971 | [-1.91, 1.09] | 0.04 |
|  | 6 | .565 | .502 | .871 | [-0.87, 2.00] | 0.06 |
| CF 4-7(5) | 6 | .974 | .642 | .654 | -[0.86, 2.81] | 0.08 |
| CF 8-12+ (6) | - | - | - | - | - | - |

*Dependent variable: CPS Delivery. Items in bold represent statistically significant findings.*

*Note.* CF- Breast = child was combination fed; breast indicates the number of months the child received breast milk; mo= months; BF = exclusive breastfeeding*.*

|  |  |  |  |  |  |  |
| --- | --- | --- | --- | --- | --- | --- |
| FF (1) | 2 | .570 | 1.325 | .998 | [-3.21, 4.35] | 0.04 |
|  | 3 | -1.992 | .567 | **.006** | [-3.61, -.37] | **0.28** |
|  | 4 | 1.061 | .657 | .588 | [-.81, 2.93] | 0.13 |
|  | 5 | -.604 | 1.091 | .994 | [-3.72, 2.51] | 0.04 |
|  | 6 | .635 | 1.036 | .990 | [-2.32, 3.59] | 0.05 |
| BF 9-12(2) | 3 | -2.562 | 1.333 | .389 | [-6.36, 1.24] | 0.15 |
|  | 4 | .491 | 1.373 | .999. | [-3.43-, 1.24] | 0.02 |
|  | 5 | -1.174 | 1.626 | .979 | [-5.81, 3.47] | 0.06 |
|  | 6 | .065 | 1.590 | 1.000 | [-4.47, 3.47] | 0.003 |
| BF 12+(3) | 4 | -3.053 | .672 | **< .001** | [1.14, 4.97] | **0.36** |
|  | 5 | -1.388 | 1.100 | .806. | [-1.75, 4.53] | 0.09 |
|  | 6 | 2.627 | 1.046 | .121 | [-.36, 5.61] | 0.20 |
| CF 0-3(4) | 5 | 1.665 | 1.149 | .697 | [-4.94, 1.61] | 0.11 |
|  | 6 | -.426 | 1.097 | .999 | [-3.56, 2.70] | 0.03 |
| CF 4-7(5) | 6 | -1.239 | 1.401 | .950 | -[-2.76, 5.24] | 0.06 |
| CF 8-12+ (6) | - | - | - | - | - | - |

**Table S3. Between-group comparisons and effect sizes for feeding method and SSQ scores**

*Dependent variable: SSQSum. Items in bold represent statistically significant findings.*

*Note.* CF- Breast = child was combination fed; breast indicates the number of months the child received breast milk; mo= months; BF = exclusive breastfeeding*.*

**References**

UNICEF. (2019). *Breastfeeding in the UK*.

<https://www.unicef.org.uk/babyfriendly/about/breastfeeding-in-the-uk/>
